# Infection Rate and Risk Factors of SARS-CoV-2 Infection in Retail Workers at the Onset of the COVID-19 Pandemic, Quebec, Canada

**DOI:** 10.3390/idr16060098

**Published:** 2024-12-16

**Authors:** Kim Santerre, Mathieu Thériault, Nicholas Brousseau, Marc-André Langlois, Corey Arnold, Joelle N. Pelletier, Caroline Gilbert, Jean-François Masson, Mariana Baz, Denis Boudreau, Sylvie Trottier

**Affiliations:** 1 Infectious Diseases Research Center of Laval University, CHU de Québec-Université Laval (CHUL Hospital), Quebec City, QC G1V 4G2, Canada; kim.santerre.1@ulaval.ca (K.S.); mathieu.theriault@crchudequebec.ulaval.ca (M.T.); caroline.gilbert@crchudequebec.ulaval.ca (C.G.); mariana.baz@chudequebec.ulaval.ca (M.B.); 2 Biological Risks Department, Institut National de Santé Publique du Québec, Quebec City, QC G1V 5B3, Canada; nicholas.brousseau@inspq.qc.ca; 3 Department of Biochemistry, Microbiology and Immunology, Faculty of Medicine, Université d’Ottawa, Ottawa, ON K1H 8M5, Canada; langlois@uottawa.ca (M.-A.L.); carnold3@uottawa.ca (C.A.); 4 Department of Chemistry, Université de Montréal, Montreal, QC H2V 0B3, Canada; joelle.pelletier@umontreal.ca (J.N.P.); jf.masson@umontreal.ca (J.-F.M.); 5 Department of Biochemistry, Université de Montréal, Montreal, QC H2V 0B3, Canada; 6 The Québec Network for Research on Protein Function, Engineering and Applications (PROTEO), Quebec City, QC G1V 0A6, Canada; 7 Département de Microbiologie-Infectiologie et d’Immunologie, Faculté de Médecine, Université Laval, Quebec City, QC G1V 0A6, Canada; 8 Quebec Center for Advanced Materials, Université de Montréal, Montreal, QC H3C 3J7, Canada; 9 Regroupement Québécois sur les Matériaux de Pointe, Université de Montréal, Montreal, QC H3C 3J7, Canada; 10 Centre Interdisciplinaire de Recherche sur le Cerveau et l’Apprentissage, Université de Montréal, Montreal, QC H3C 3J7, Canada; 11 Institut Courtois, Université de Montréal, Montreal, QC H2V 0B3, Canada; 12 Département de Chimie et Centre d’Optique, Photonique et Laser (COPL), Université Laval, Quebec City, QC G1V 0A6, Canada; denis.boudreau@chm.ulaval.ca

**Keywords:** COVID-19, SARS-CoV-2, cohort, food workers, retail workers, serology

## Abstract

**Background/Objectives:** During the pandemic, client-facing workers were perceived to be at greater risk of SARS-CoV-2 infection. This study investigated the risk factors for SARS-CoV-2 infection among a cohort of 304 retail workers in the Quebec City metropolitan area. **Methods:** After providing consent, participants were interviewed to gather information on demographic, socioeconomic, behavioural, and occupational variables. They were subsequently followed for up to five visits, scheduled every 12 ± 4 weeks. The study covered critical periods before and during the emergence of the Omicron variants and included retrospective reporting of COVID-19 symptoms and virus detection tests to capture the pandemic’s early stages. **Results:** During the observation period, 173 (57%) participants experienced a first episode of COVID-19. Serological evidence of recent infection was detected in 160 participants (53%), while 117 (38%) reported a positive virus detection test. In adjusted analyses, risk factors for infection included younger age, a diagnosis of lung disease, longer weekly working hours, more frequent social gatherings, and having received fewer than three doses of vaccine. Notably, the increased risk associated with younger age and longer working hours was observed only after the relaxation of public health measures in the spring of 2022. **Conclusions:** These data suggest that during the early years of the pandemic when strict public health measures were in place, retail work was not a significant risk factor for SARS-CoV-2 infection in Quebec City metropolitan area. These findings highlight the complex dynamics of COVID-19 transmission and the effectiveness of workplace protective measures.

## 1. Introduction

In studies carried out early in the SARS-CoV-2 pandemic, workers with client-facing occupations were perceived to be at greater risk of infection than those working remotely. Client-facing workers included health care workers (HCWs) [1,2], but also individuals employed in the retail and food service sectors who directly interacted with clients. The potential risk was supported by several serological studies [3,4,5,6,7]. For example, a serosurvey in New York City reported a higher seroprevalence of anti-spike antibodies among grocery store and restaurant workers compared to most subgroups of HCWs [3]. Another serosurvey conducted in Switzerland reported an above-average seroprevalence in kitchen staff and grocery store workers compared to other essential workers [6]. In the Netherlands, individuals working in the hospitality sector were more likely to have a positive PCR test result than those working in non-close-contact occupations [7]. In Japan, restaurants and bars were the second most common setting of SARS-CoV-2 outbreaks after health care facilities [8,9]. However, a recent UK study suggests that retail and hospitality workers were not at increased risk of a SARS-CoV-2 infection [10].

In this study, we measured SARS-CoV-2 exposure among restaurant/bar and grocery and hardware store workers in Quebec, the Canadian province with the highest mortality rate due to this virus during the early phase of the pandemic [11,12]. Consequently, we set up a longitudinal cohort to identify the infection rate and risk factors for SARS-CoV-2 infection among retail workers over the course of the very first years of the pandemic and to measure the dynamic of infection in this exposed group based on the demographic, occupational, and residential characteristics of the participants.

## 2. Materials and Methods

### 2.1. Study Design, Participants, and Detection of SARS-CoV-2 Infection

A prospective cohort of food and retail (restaurant/bar and grocery and hardware store) workers with public-facing roles was recruited using different outreach strategies including online and email invitations. Exclusion criteria included previous hospitalization for SARS-CoV-2 [13]. Participants were followed over a period of 18 months (20 April 2021 to 3 October 2022) with up to five visits every 12 ± 4 weeks. Each participant was in the study for a maximum of 48 weeks. Briefly, participants answered a questionnaire based on the Canadian Immunity Task Force (CITF) Standardized Core Survey Data Elements form [14]. Included were questions about type of work, age, sex, education level, weight, comorbidities, smoking habits, influenza vaccine status, work region, weekly working hours, social gatherings, travel, and number of people in the household [13]. Participant demographics are explained in detail in our related publication [13].

Information was collected on any SARS-CoV-2 detection test (PCR or rapid antigen) performed from the start of the pandemic (February 2020) to the last visit, and on the presence of symptoms related to COVID-19, such as cough, fever, shortness of breath, sore muscles, headache, sore throat, diarrhea, runny nose, and decreased sense of smell or taste. Information on the type of COVID-19 vaccine and the date and number of doses received was also collected. Blood samples were collected at each visit to assess antibody response to SARS-CoV-2 by enzyme-linked immunosorbent assays (ELISAs). These ELISAs were developed at the Universities of Ottawa and Toronto and were calibrated against a World Health Organization reference standard [15,16,17]. Serological evidence of recent SARS-CoV-2 infection was determined by the combined detection of IgG antibodies against spike and nucleocapsid proteins.

### 2.2. Study Exposures and Outcome

Serologic analysis was intended to identify infections, diagnosed or not, while virus detection tests on nasopharyngeal samples captured infections suspected by the participants. Both tests were deemed valid, and an infection was diagnosed if at least one of these two tests was positive. An infection was considered symptomatic when the participant reported at least one symptom related to COVID-19. Analysis focused on the occurrence of the first diagnosis of infection during the study.

### 2.3. Sample Size and Statistical Analysis

The study was designed at the onset of the COVID-19 pandemic, before the first COVID-19 vaccine was approved, as a prospective cohort to investigate the immune response to SARS-CoV-2. At the time, the estimated infection rate in our population was 2% in the absence of vaccination. The original goal was to collect samples from 12 infected volunteers, with a target enrolment of 600 participants. As the rate of COVID-19 increased to reach more than 100 infected participants, enrolment was halted at 304 participants.

Kaplan–Meier curves were used to illustrate the cumulative event rates of primary infections. To identify risk factors for SARS-CoV-2 infection, adjusted (multivariate) hazard ratios were calculated using the Cox regression analysis. Univariate analyses identified variables associated with SARS-CoV-2 infection that we included in the multivariate analyses. All statistical analyses were performed using R software (2024.09.1 Build 394, Posit Software PBC, Boston, MA, USA), with a significance threshold set at 5%, and all tests were two-sided.

### 2.4. Confidentiality and Data Storage

A unique, anonymized identifier was assigned to each participant and used to identify the samples and data. The samples will be stored for up to 10 years, and the data for at least 15 years using REDCap electronic data capture tools hosted at the Centre de Recherche du CHU de Québec—Université Laval [18,19].

### 2.5. Patient and Public Involvement Statement

No public stakeholders were involved in establishing or designing this cohort. This study was approved by the research ethics committee of the « CHU de Québec—Université Laval » (registration number 2021-5744).

## 3. Results

### 3.1. Participant Characteristics

**Figure 1:**
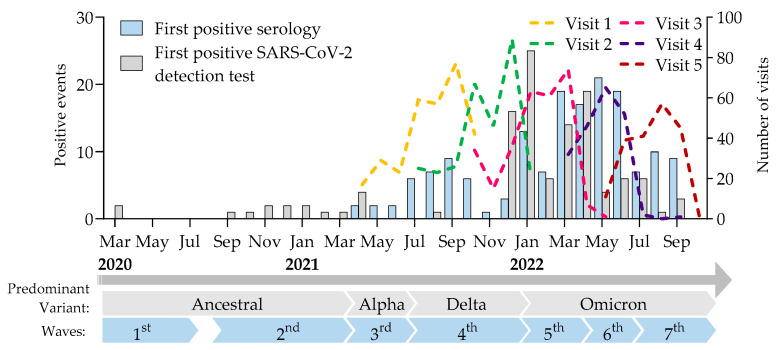
Timeline of the study illustrating the visits (coloured dashed lines) and the first occurrences of seropositivity (blue bars, combined detection of IgG antibodies against spike and nucleocapsid proteins) and of SARS-CoV-2 positive virus detection (grey bars, PCR or rapid antigen). Adapted from [13].

A total of 304 individuals were initially recruited between April 2021 and May 2022, including restaurant/bar (49.0%), grocery store (36.8%), and hardware store (14.1%) workers (Figure 1). Following the emergence of the Omicron variant, 198 participants completed two additional visits from March to October 2022. The average age of the cohort was 41.3 years, and 57.9% were female. In terms of education, 39.4% had a high school diploma, 33.2% held a college certificate, and 22.7% had a university degree.

Regarding body mass index (BMI), 41.1% of participants had a healthy weight, 27.0% were overweight, and 30.6% were obese. Around 31.9% reported at least one comorbidity, including 33 individuals with lung disease and 35 with arterial hypertension. Smoking was reported by 17.4% of participants, while 7.9% used e-cigarettes. Only 13.8% had received influenza vaccine within the previous year.

The majority of participants (78.9%) worked in the Quebec City (Capitale-Nationale) region. About 46.4% worked more than 30 h per week. Additionally, 40.8% attended more than ten large social gatherings (with ten or more people), and 47.0% reported traveling outside of the province during the study period. In terms of living arrangements, 61.8% lived alone or with one other person, 30.9% lived with two to three coresidents, and 7.2% lived with more than three coresidents.

### 3.2. Detection of SARS-CoV-2 Infection

**Figure 2:**
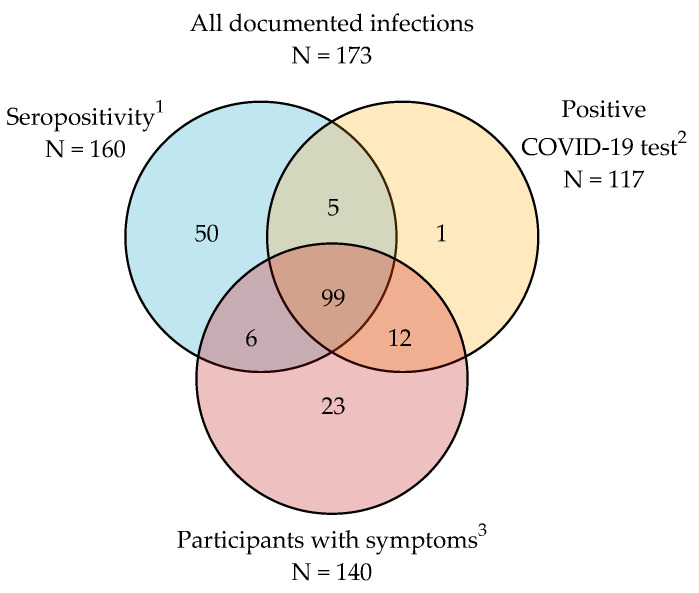
Documented infections and participants with symptoms. ^1^ Combined detection of IgG antibodies against spike and nucleocapsid proteins; ^2^ nasopharyngeal PCR or rapid antigen test; ^3^ at least one symptom related to COVID-19: cough, fever, shortness of breath, sore muscles, headache, sore throat, diarrhea, runny nose, or decreased sense of smell or taste.

Throughout the study, 117 (38.5%) participants reported at least one diagnosis of COVID-19 as determined by virus detection (40 nasopharyngeal PCR and 77 rapid antigen tests) (Figure 2). Serological evidence of SARS-CoV-2 new infection (combined detection of IgG antibodies against spike and nucleocapsid proteins) was found in 160 (53%) of the 304 participants. Of those seropositive participants, 104 (65%) reported a positive virus detection test, while 56 (35%) showed serological evidence of new infection with negative or no virus detection test. Of the 117 participants with a positive virus detection test, 104 (89%) showed serological evidence of a new SARS-CoV-2 infection, and 13 (11%) were never detected through serological analysis.

Infection, symptomatic or not, was diagnosed in a total of 173 (57%) participants based on the presence of antibodies against SARS-CoV-2 or from a positive reported virus detection test. Of these, 117 (68%) experienced at least one symptom of COVID-19 [13], including 111 participants reporting positive virus detection tests and 6 who did not undergo virus detection testing and were detected by serology only. Infection was documented in 56 (32%) participants who did not report symptoms. None of the participants had an infection severe enough to require hospitalization at baseline or during the study.

### 3.3. SARS-CoV-2 Vaccine Uptake

Vaccination of the general population began in the spring of 2021. The vaccines available during the study, approved by Canadian health authorities, included the monovalent Comirnaty (Pfizer–BioNTech), Spikevax (Moderna), and Vaxzevria (AstraZeneca), each requiring two doses to complete the primary series. By winter 2022, public health authorities recommended a third dose as a booster. At enrolment, 62.2% of the participants had already received two doses of the COVID-19 vaccine. Among the participants, 286 (94.1%) completed the primary series, and 152 (50.0%) received at least one booster dose. To evaluate the overall impact of vaccination, participants were divided into two groups: those who received up to two doses, and those who received three or more doses. While this approach did not allow for measurement of vaccine effectiveness, it provided a general understanding of the benefits of greater adherence to vaccination campaigns over the study period.

### 3.4. Cumulative Events of SARS-CoV-2 Infection

**Figure 3:**
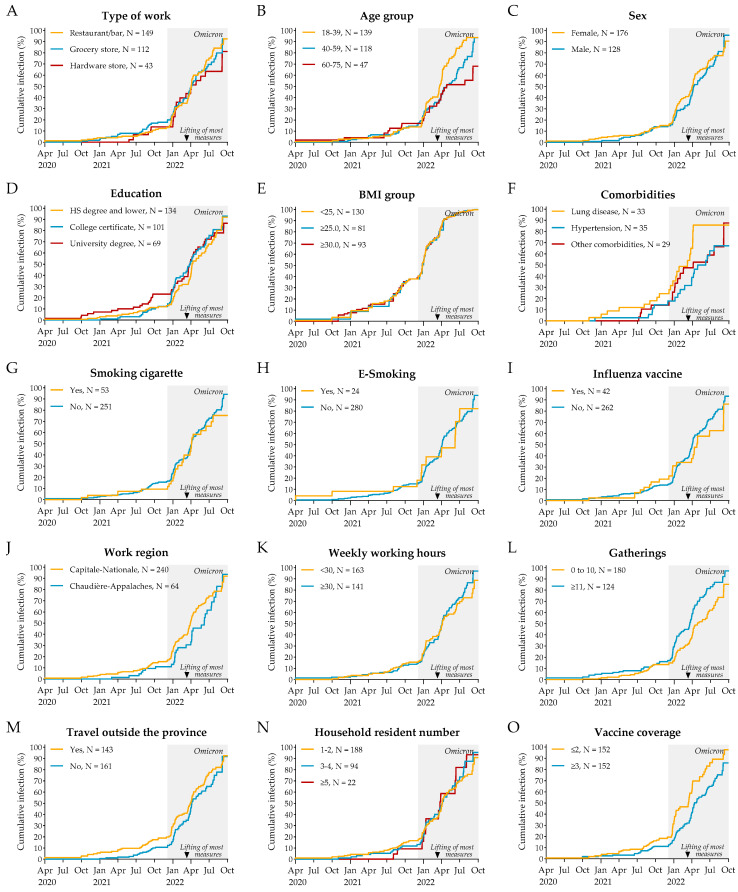
Kaplan–Meier curves depicting the cumulative event rate of all infections, stratified by type of work (**A**), age group (**B**), sex (**C**), education level (**D**), BMI group (**E**), comorbidities (**F**), smoking habits (**G**,**H**), influenza vaccine status (**I**), work region (**J**), weekly working hours (**K**), social gatherings frequency (**L**), travel frequency (**M**), household resident number (**N**), and number of COVID-19 vaccinations received (**O**). The shaded area indicates the Omicron period. The arrow indicates the lifting of most public health measures, which occurred on 12 March 2022.

As shown by the Kaplan–Meier curves, the rate of infection remained low during the initial phase of the study but increased with the emergence of the Omicron variant (Figure 3). A notable trend was observed starting mid-March 2022, with higher infection rates among younger individuals (Figure 3B), those with comorbidities (Figure 3F), and those who attended more social gatherings (Figure 3L), and those with fewer than three COVID-19 vaccine doses (Figure 3O). This period coincided with the lifting on 12 March 2022 of most public health measures, which had been implemented in March 2020 and enforced, either partially or fully, throughout most of the study period [20]. To further investigate this, we stratified the Cox regression analyses before and after 12 March 2022, accounting for this significant event.

Crude Cox regression analyses identified several significant associations between SARS-CoV-2 infection and specific risk factors (Table 1). Age was a key factor, with individuals aged 40–59 and 60–75 having a lower risk of infection compared to younger participants (HR = 0.65 and 0.47, respectively). This association was particularly important after public health measures were relaxed (HR = 0.41 and 0.19, respectively). Participants with lung disease had an increased risk of COVID-19 infection, especially before the relaxation of measures (HR = 1.89). Working longer hours (≥30 h per week) was also associated with a higher risk of infection, but only after the relaxation of public health measures (HR = 1.63). Additionally, attending gatherings of more than ten people consistently increased the risk of infection (HR = 1.68), and the association was more important after public health restrictions were lifted (HR = 1.88). Vaccination played a protective role, with individuals who had received three or more doses of COVID-19 vaccine demonstrating a significantly lower risk of infection across all time periods (HR = 0.56). In contrast, other factors such as type of work, sex, education level, BMI, smoking, workplace region, travel, and number of residents in the household did not show significant differences in the risk of infection.

Adjusted Cox regression analyses were performed to account for potential confounding factors (Table 2). Factors associated with SARS-CoV-2 infection remained similar to the crude analysis. Older age remained a strong and significant protective factor regarding SARS-CoV-2 infection risk, especially after relaxation of public health measures (HR = 0.20 and 0.22, respectively, for 40–59- and 60–75-year-olds). Lung disease continued to show a significant association with infection risk (HR = 2.06). Vaccination maintained its protective association, with individuals receiving three or more doses showing a lower risk of infection throughout the study (HR = 0.61). Interestingly, the number of working hours was identified as one of the strongest risk factor in the adjusted analyses, with individuals working 30 h or more per week showing a significant increase in risk of infection, but only after the relaxation of measures (HR = 3.21). Finally, attending gatherings of more than ten people was associated with a higher risk of infection when considering the whole study period (HR = 1.42).

## 4. Discussion

This study investigated the infection rate and risk factors for COVID-19 among a cohort of 304 retail (restaurant/bar and grocery and hardware store) workers in the Quebec City metropolitan area. Conducted at the beginning of the pandemic, it spanned critical periods before and during the emergence of the highly contagious Omicron variants. Public health measures implemented at the beginning of the pandemic were lifted about 6 months before the end of the study. The study included both prospective and retrospective reporting by the participants in positive virus detection tests (PCR or rapid antigen) and covered the pandemic’s initial stages. Serological surveillance of infection in each participant was done by detection of IgG antibodies against spike and nucleocapsid proteins in up to five serial blood samples.

Participants were recruited from three different types of retail stores, with the hypothesis that the risk would differ from one type of work to another. Grocery and hardware stores were considered essential services and remained open throughout the pandemic. Public health measures were enforced and generally well respected. The difference between the two lay in customer interaction patterns: grocery stores typically serve a large number of clients in many brief interactions, whereas hardware store workers tend to assist fewer customers, often providing advice, leading to longer interactions. In contrast, restaurants and bars experienced intermittent opening and closure during the same period. Public health measures were also more challenging to enforce in these settings due to the inherently social nature of this sector of activity and its primary purpose—the consumption of food and beverages—which made continuous mask-wearing impossible.

Overall, the risk of COVID-19 infection varied little by type of work, with no statistical differences observed. This consistency may be attributed to the effectiveness of protective measures at work, suggesting that most infections resulted from community transmission rather than workplace exposure. This is also reflected by the fact that the infection rate in our cohort was similar to that observed in the general population from the start of the pandemic up to 4 December 2021, when infection was detected by monitored PCR testing [21]. Beginning 5 January 2022, PCR testing was restricted to certain priority groups and was replaced by self-administered rapid antigen detection tests. Because of the unsupervised nature of this testing, they were not systematically recorded, limiting our ability to perform comparisons from this point on. However, seroprevalence monitoring showed a similar increase of acute SARS-CoV-2 infection within the general Canadian population following the emergence of Omicron, as determined by the presence of nucleocapsid protein antibodies [22]. Overall, this suggests that food and retail workers were not at significantly increased risk of SARS-CoV-2 infection due to their occupational status.

Interestingly, while no difference was observed between types of work, participants working full time exhibited a significantly higher infection rate compared to part-time workers. Although this may be due to the increased risk associated with longer workplace exposure, this elevated risk was only observed toward the end of the study, coinciding with the relaxation of COVID-19 protective measures, highlighting the efficacy of public health measures to limit transmission of this virus.

Older participants exhibited a lower rate of infection, which is contrary to a study carried out in Kosovo, but in line with data from Brazil and from a seroprevalence study of the Canadian population [22,23,24]. The reduced risk could also be attributed to higher vaccination rates and stricter adherence to protective measures among older individuals, who perceived themselves as more vulnerable. Additionally, our participants might represent a subgroup of still-working 60–75-year-old individuals who have better overall health and may be less susceptible to infection than the average population in this age group. However, a behavioural factor linked to age may also play a role, with younger participants potentially engaging in higher-risk activities, as our data indicate that infection rates were similar across age groups during the enforcement of public health measures, with the difference emerging only after these measures were relaxed. Similarly, we believe that our participants with higher BMI were not at higher risk of infection as they were actively working, did not have prior COVID-19 hospitalization, and most of them were fully vaccinated.

The increased risk among those with lung disease aligns with studies showing that individuals with underlying lung conditions were more likely to be seropositive than those without such a condition [25,26]. The association between smoking and risk of SARS-CoV-2 infection is controversial, and our data suggest that smoking, either cigarettes or vaping, is not a risk factor for catching a SARS-CoV-2 infection [27,28]. The rate of infection was higher among participants who travelled during the study period. However, this association did not reach statistical significance when confounders were accounted for, suggesting that other factors might contribute to the increased risk. As expected, attending more social gatherings was also a risk factor for infection in the adjusted analyses.

In this closely monitored cohort, 32.4% of participants were found to have asymptomatic infection, which is consistent with a meta-analysis reporting a pooled prevalence of 32.4% among individuals infected with SARS-CoV-2 Omicron variants [29]. A small number (29) of participants with symptoms did not undergo virus detection testing, and of those, only 21% had serological evidence of SARS-CoV-2 infection. This suggests that other respiratory viruses may have been circulating and causing respiratory symptoms during the pandemic.

Our participants were highly vaccinated, with half receiving three or more doses during the study. Overall, a higher number of vaccine doses was statistically associated with a reduced infection risk, particularly during the Omicron period, as the third dose was administered at the beginning of this phase. However, our ability to measure true vaccine effectiveness was limited by several potential biases, such as loss to follow-up and variations in vaccination timing. Additionally, participants who were more inclined to get vaccinated may have also been more cautious of infection risks and may have taken additional protective measures that were not accounted for in this study.

In summary, our findings indicate that retail workers in the province of Quebec did not experience a significantly increased risk of SARS-CoV-2 infection due to their specific occupational status during the pandemic. The variations in observed risk levels among participants were primarily driven by the external elements that occurred during the studied periods, including the emergence of Omicron variants, the third vaccination campaign, and the gradual relaxation of protective measures.

### Strengths and Limitations

Our study had several strengths, including the use of both serological and virus detection diagnostic methods to capture a comprehensive view of infection rate and the application of statistical analysis to control confounding variables. However, there are notable limitations to consider. First, reliance on self-reported data for symptoms and testing may have introduced recall bias. As serology data helped to identify cases, and given the exceptional nature of the pandemic, it is unlikely that a positive COVID-19 diagnosis was overlooked. Second, concerns about losing work might have led to underreporting of SARS-CoV-2 infections. However, the impact of lost work and income was mitigated thanks to the Canada’s universal health care coverage, which included testing and vaccination resources, and emergency benefits provided to those who needed to isolate due to COVID-19 [30]. Therefore, disparities in access to health care and resources were not anticipated to significantly affect the measured susceptibility to SARS-CoV-2.

The initial study was planned to consist of three visits, concluding in April 2022. However, with the emergence of the Omicron variant, two additional visits were initiated to gather further data. Of the 304 participants, 198 were recruited in this extended context, allowing us to monitor infections until October 2022. This extension introduced a potential loss to follow-up bias, which may have affected the results depending on the categories of participants who remained in the study versus those who did not. To address this, we used Kaplan–Meier and Cox regression analyses, as these methods account for loss to follow-up.

In this study, vaccine coverage was high; by the time the Omicron variant emerged, nearly 95% of participants had completed their primary vaccination series, and half had received a first booster dose. This extensive coverage limited our ability to assess the protective impact of the primary vaccine series. The high vaccination rate likely reflected retail workers’ perceived higher risk of SARS-CoV-2 exposure compared to the general population, motivating them to prioritize vaccination to reduce infection risk. However, it is important to consider the potential for selection bias; individuals willing to participate in research might also be more likely to have received the vaccine, which could skew the sample toward those who are more proactive about health measures.

## 5. Conclusions

This study demonstrates that in the first years of the pandemic, client-facing retail workers of the Quebec City metropolitan area were at a similar risk of SARS-CoV-2 infection to the general Canadian population, despite differences of exposure in their type of work. This suggests that protective measures at work were effective, and that transmission was driven by other variables, as demonstrated by the increased infection rate in some risk groups after the relaxation of public health measures. Overall, these findings acknowledge the importance of community-level interventions and continued adherence to protective measures to mitigate the risk of COVID-19 infection, irrespective of professional occupation.

## Figures and Tables

**Table 1:** Crude Cox regression analyses of risk factors for SARS-CoV-2 infection.

	Whole Study Period		Before Measures Relaxation ^1^		After Measures Relaxation ^1^	
Characteristic	Crude HR (95% CI)	*p*-Value	Crude HR (95% CI)	*p*-Value	Crude HR (95% CI)	*p*-Value
**Type of work**						
Grocery store	Ref.		Ref.		Ref.	
Resto/Bar	1.02 (0.74–1.41)	0.908	0.84 (0.54–1.30)	0.426	1.30 (0.80–2.11)	0.297
Hardware store	0.82 (0.51–1.33)	0.426	1.01 (0.55–1.82)	0.986	0.59 (0.26–1.37)	0.220
**Age (years)**						
18–39	Ref.		Ref.		Ref.	
40–59	0.65 (0.47–0.90)	0.010	0.89 (0.58–1.38)	0.612	0.41 (0.25–0.67)	<0.001
60–75	0.47 (0.29–0.75)	0.002	0.90 (0.50–1.61)	0.712	0.19 (0.089–0.43)	<0.001
**Sex**						
Female	Ref.		Ref.		Ref.	
Male	0.92 (0.68–1.24)	0.570	0.80 (0.53–1.20)	0.282	1.09 (0.69–1.71)	0.720
**Education**						
High school	Ref.		Ref.		Ref.	
College	1.19 (0.84–1.68)	0.327	1.40 (0.88–2.23)	0.150	0.97 (0.57–1.64)	0.902
University	1.16 (0.79–1.71)	0.447	1.43 (0.85–2.40)	0.177	0.91 (0.51–1.63)	0.748
**BMI**						
<25 (healthy weight)	Ref.		Ref.		Ref.	
≥25 (overweight)	0.77 (0.53–1.13)	0.189	0.75 (0.45–1.26)	0.282	0.81 (0.46–1.44)	0.473
≥30 (obese)	1.08 (0.76–1.52)	0.669	0.97 (0.61–1.54)	0.909	1.23 (0.73–2.09)	0.435
**Comorbidities**						
No	Ref.		Ref.		Ref.	
Lung disease ^2^	1.69 (1.05–2.71)	0.028	1.89 (1.07–3.33)	0.028	1.44 (0.61–3.38)	0.408
Arterial hypertension ^3^	0.74 (0.45–1.20)	0.216	0.90 (0.46–1.76)	0.760	0.59 (0.29–1.20)	0.145
Other ^4^	0.93 (0.56–1.56)	0.792	1.46 (0.79–2.72)	0.232	0.49 (0.19–1.22)	0.123
**Cigarette smoking**						
No	Ref.		Ref.		Ref.	
Yes	0.88 (0.58–1.33)	0.539	0.98 (0.57–1.67)	0.931	0.76 (0.39–1.47)	0.408
**E-smoking**						
No	Ref.		Ref.		Ref.	
Yes	0.99 (0.54–1.83)	0.980	1.14 (0.53–2.46)	0.743	0.81 (0.29–2.23)	0.682
**Influenza vaccine ^5^**						
No	Ref.		Ref.		Ref.	
Yes	0.85 (0.54–1.33)	0.474	1.01 (0.56–1.81)	0.976	0.69 (0.34–1.38)	0.290
**Workplace region**						
Capitale-Nationale	Ref.		Ref.		Ref.	
Chaudière-Appalaches	0.78 (0.53–1.14)	0.204	0.70 (0.41–1.20)	0.200	0.88 (0.51–1.50)	0.631
**Working hours**						
<30 h/w	Ref.		Ref.		Ref.	
≥30 h/w	1.15 (0.85–1.55)	0.364	0.88 (0.59–1.32)	0.542	1.63 (1.02–2.59)	0.041
**Gatherings ^6^**						
≤10	Ref.		Ref.		Ref.	
>10	1.681 (1.24–2.27)	0.001	1.54 (1.03–2.30)	0.035	1.88 (1.20–2.96)	0.006
**Travel**						
No	Ref.		Ref.		Ref.	
Yes ^7^	1.27 (0.94–1.71)	0.127	1.39 (0.93–2.07)	0.112	1.12 (0.71–1.78)	0.618
**Number of household residents**						
1–2	Ref.		Ref.		Ref.	
3–4	1.09 (0.78–1.53)	0.612	1.02 (0.65–1.58)	0.939	1.20 (0.71–2.02)	0.498
≥5	1.23 (0.68–2.24)	0.491	0.90 (0.39–2.09)	0.808	1.89 (0.81–4.45)	0.142
**Number of COVID-19 vaccinations**						
0–2	Ref.		Ref.		Ref.	
≥3	0.56 (0.41–0.77)	<0.001	0.58 (0.38–0.87)	0.009	0.53 (0.32–0.87)	0.012

CI = confidence interval, Ref. = reference. ^1^ Relaxation of most COVID-19 prevention measures on 12 March 2022. ^2^ Lung disease: asthma and chronic pulmonary disease. ^3^ Excluding lung disease. ^4^ Other comorbidities: diabetes mellitus, hypothyroidism, cancer, cardiovascular disease, immunodeficiency, chronic neurological disorder, liver disease, blood disorders, obesity, and kidney disease. ^5^ Influenza vaccine within the year prior to the first visit. ^6^ Gathering is defined as a meeting of a group of at least ten individuals during the study. ^7^ Travel outside the province of Quebec.

**Table 2:** Adjusted Cox regression analyses of risk factors for SARS-CoV-2 infection.

	Whole Study Period		Before Measures Relaxation ^1^		After Measures Relaxation ^1^	
Characteristic	Adjusted HR (95% CI)	*p*-Value	Adjusted HR (95% CI)	*p*-Value	Adjusted HR (95% CI)	*p*-Value
**Type of work**						
Grocery store	Ref.		Ref.		Ref.	
Resto/Bar	0.91 (0.62–1.34)	0.636	0.71 (0.42–1.19)	0.196	1.3 (0.69–2.44)	0.419
Hardware store	0.86 (0.51–1.46)	0.586	0.99 (0.53–1.87)	0.982	0.56 (0.21–1.53)	0.259
**Age (years)**						
18–39	Ref.		Ref.		Ref.	
40–59	0.57 (0.39–0.85)	0.005	0.97 (0.58–1.61)	0.892	0.2 (0.1–0.39)	<0.001
60–75	0.58 (0.32–1.05)	0.070	1.18 (0.56–2.48)	0.658	0.22 (0.08–0.59)	0.002
**Sex**						
Female	Ref.		Ref.		Ref.	
Male	0.85 (0.61–1.17)	0.310	0.83 (0.54–1.29)	0.413	0.63 (0.36–1.1)	0.105
**Education**						
High school	Ref.		Ref.		Ref.	
College	1.11 (0.76–1.61)	0.598	1.33 (0.81–2.19)	0.259	0.99 (0.53–1.83)	0.967
University	1.25 (0.81–1.92)	0.320	1.51 (0.85–2.71)	0.162	1.01 (0.5–2.04)	0.984
**BMI**						
<25 (healthy weight)	Ref.		Ref.		Ref.	
≥25 (overweight)	0.84 (0.55–1.28)	0.418	0.75 (0.43–1.31)	0.313	0.84 (0.42–1.68)	0.620
≥30 (obese)	1.18 (0.8–1.73)	0.397	1.01 (0.6–1.69)	0.971	1.55 (0.82–2.95)	0.181
**Comorbidities**						
No	Ref.		Ref.		Ref.	
Lung disease ^2^	2.06 (1.23–3.44)	0.006	1.86 (1.01–3.42)	0.047	2.24 (0.77–6.54)	0.138
Arterial hypertension ^3^	1.04 (0.59–1.83)	0.903	1.13 (0.53–2.44)	0.746	1.13 (0.47–2.73)	0.779
Other ^4^	1.4 (0.78–2.52)	0.253	1.87 (0.91–3.82)	0.086	0.78 (0.27–2.27)	0.649
**Cigarette smoking**						
No	Ref.		Ref.		Ref.	
Yes	0.9 (0.58–1.41)	0.653	1.12 (0.63–1.97)	0.706	0.64 (0.3–1.35)	0.237
**E-Smoking**						
No	Ref.		Ref.		Ref.	
Yes	0.84 (0.42–1.69)	0.622	1.09 (0.46–2.6)	0.841	0.57 (0.16–1.99)	0.379
**Influenza vaccine ^5^**						
No	Ref.		Ref.		Ref.	
Yes	1.07 (0.66–1.72)	0.794	1.08 (0.58–2.01)	0.812	1.24 (0.56–2.73)	0.595
**Workplace region**						
Capitale-Nationale	Ref.		Ref.		Ref.	
Chaudière-Appalaches	0.68 (0.45–1.03)	0.071	0.73 (0.42–1.28)	0.275	0.36 (0.18–0.72)	0.004
**Working hours**						
<30 h/w	Ref.		Ref.		Ref.	
≥30 h/w	1.53 (1.04–2.24)	0.029	1.02 (0.62–1.68)	0.940	3.21 (1.63–6.32)	0.001
**Gatherings ^6^**						
≤10	Ref.		Ref.		Ref.	
>10	1.42 (1–2.01)	0.048	1.55 (0.97–2.48)	0.066	1.2 (0.67–2.17)	0.535
**Travel**						
No	Ref.		Ref.		Ref.	
Yes ^7^	0.99 (0.7–1.39)	0.936	1.12 (0.71–1.75)	0.628	0.64 (0.35–1.16)	0.142
**Number of household residents**						
1–2	Ref.		Ref.		Ref.	
3–4	0.96 (0.67–1.39)	0.836	0.94 (0.58–1.53)	0.812	0.94 (0.53–1.67)	0.828
≥5	1.21 (0.62–2.37)	0.584	0.98 (0.39–2.48)	0.973	1.96 (0.64–5.97)	0.237
**Number of COVID-19 vaccinations**						
0–2	Ref.		Ref.		Ref.	
≥3	0.61 (0.43–0.87)	0.006	0.55 (0.35–0.88)	0.012	0.47 (0.25–0.89)	0.021

CI = confidence interval, Ref. = reference. ^1^ Relaxation of most COVID-19 prevention measures on 12 March 2022. ^2^ Lung disease: asthma and chronic pulmonary disease. ^3^ Excluding lung disease. ^4^ Other comorbidities: diabetes mellitus, hypothyroidism, cancer, cardiovascular disease, immunodeficiency, chronic neurological disorder, liver disease, blood disorders, obesity, and kidney disease. ^5^ Influenza vaccine within the year prior to the first visit. ^6^ Gathering is defined as a meeting of a group of at least ten individuals during the study. ^7^ Travel outside the province of Quebec.

## Data Availability

More detailed, participant-level information is publicly available on an online platform developed by Maelstrom Research [31]. Researchers with other enquiries or collaboration proposals may contact Sylvie Trottier—the principal investigator in charge of setting up the cohort—at sylvie.trottier@crchudequebec.ulaval.ca.
